# Design, Synthesis, and Structure-Property Relationships of Er^3+^-Doped TiO_2_ Luminescent Particles Synthesized by Sol-Gel

**DOI:** 10.3390/nano8010020

**Published:** 2018-01-02

**Authors:** Pablo Lopez-Iscoa, Diego Pugliese, Nadia G. Boetti, Davide Janner, Giovanni Baldi, Laeticia Petit, Daniel Milanese

**Affiliations:** 1 Dipartimento di Scienza Applicata e Tecnologia (DISAT) and INSTM UdR Torino Politecnico, Politecnico di Torino, Corso Duca degli Abruzzi 24, 10129 Torino, Italy; pablo.lopeziscoa@polito.it (P.L.-I.); davide.janner@polito.it (D.J.); daniel.milanese@polito.it (D.M.); 2 Istituto Superiore Mario Boella, Via P. C. Boggio 61, 10138 Torino, Italy; boetti@ismb.it; 3 CE.RI.COL., Colorobbia Research Center, Via Pietramarina 53, 50053 Sovigliana-Vinci (FI), Italy; baldig@colorobbia.it; 4 Laboratory of Photonics, Tampere University of Technology, Korkeakoulunkatu 3, 33720 Tampere, Finland; laeticia.petit@tut.fi; 5 nLIGHT Corporation, Sorronrinne 9, 08500 Lohja, Finland; 6 IFN–CNR, CSMFO Lab., Via alla Cascata 56/C, 38123 Povo (TN), Italy

**Keywords:** erbium-doped titania, sol-gel synthesis, photoluminescence

## Abstract

Titania particles doped with various concentrations of Erbium were synthesized by the sol-gel method followed by different heat treatments. The shape and the grain growth of the particles were noticeably affected by the concentration of Erbium and the heat treatment conditions. An infrared emission at 1530 nm, as well as green and red up-conversion emissions at 550 and 670 nm, were observed under excitation at 976 nm from all of the synthesized particles. The emission spectra and lifetime values appeared to be strongly influenced by the presence of the different crystalline phases. This work presents important guidelines for the synthesis of functional Er^3+^-doped titania particles with controlled and tailored spectroscopic properties for photonic applications.

## 1. Introduction

Titania (TiO_2_) is one of the most intensively studied materials owing to a series of interesting properties, such as its semiconducting behavior, low toxicity, biocompatibility, high chemical stability, and simple and economic production [1,2,3]. All these features enable TiO_2_ to be used in a wide range of application fields, such as biomedicine, photocatalysis, and photoluminescence [4,5,6].

TiO_2_ also shows the remarkable capability to host dopants able to modify its properties. In particular, its low phonon energy (<700 cm^−1^) reduces multiphonon relaxation, thus increasing the efficiency of the luminescent processes [7,8]. Indeed, the luminescence of Er^3+^ ions at 1540 nm makes Er^3+^-doped TiO_2_ systems suitable for optical planar waveguides, lasers, and fiber amplifiers for telecommunications [8,9,10,11]. In addition, red and green up-conversion emissions [12,13] make it a promising material for an even broader range of applications, such as photovoltaics, display technologies, medical diagnostics, and solid state lasers [14,15,16,17].

The properties of a bulk material are significantly different with respect to the ones exhibited by the micro-/nano-particles [17,18,19]. Among the different synthesis methods employed for the fabrication of the TiO_2_ particles, the sol-gel synthesis method has been demonstrated to allow a reliable and precise control of particle size and morphology [20,21,22,23]. The incorporation of rare-earth ions into the TiO_2_ nano-particles has drawn noticeable interest in recent times, demonstrating the potential of this type of material [24,25]. Despite the intense research efforts, a careful analysis of the effects of Er^3+^ doping on the TiO_2_ particles properties is still lacking. Alongside this, a deeper investigation of the main factors affecting the luminescence of those particles, such as their crystalline structure and the presence of hydroxyl ions, is needed.

The presence of OH– in the structure of the material is a major inconvenience which dramatically affects its luminescence properties. A heat treatment is usually performed to remove water and hydroxyl groups (Ti–OH) and thus to achieve an increase of its lifetime values and an improvement of its fluorescence properties [26]. For the different temperatures, three regimes can be distinguished. At calcination temperatures lower than 600 °C, the OH– is desorbed after the heat treatment but partial rehydration of the sample can still occur, thus leading to a decrease in the luminescence properties [27]. For treatments at temperatures between 800 and 1000 °C, the quenching phenomena are reduced by the removal of OH– but they are still present due to the different crystalline phases and concentrations of the Er^3+^ ions in the TiO_2_ matrix [28]. Full decomposition of hydroxyl groups can be achieved by a heat treatment at ~1000 °C, although the crystalline phases obtained are rutile and pyrochlore (Er_2_Ti_2_O_7_), which are detrimental for the luminescence properties [29]. The anatase to rutile phase transition usually occurs in the range 600–1000 °C [30,31], while at temperatures between 800 and 1000 °C [8,32] and at high concentrations of Er^3+^ (7.5 mol% Er_2_O_3_ [33]), the pyrochlore phase (Er_2_Ti_2_O_7_) starts to appear. This last compound has been reported to strongly affect the luminescence, giving very short lifetime values (<<1 ms) that have been related to the quenching caused by the high concentration of Er^3+^ ions [28].

In this paper, a systematic study on TiO_2_ micro-/nano-particles synthesized by sol-gel and doped with different concentrations of Er_2_O_3_ (0.5, 2, 5, 10, and 14.3 mol%) is reported. To remove the hydroxyl groups and control the crystalline structure of the particles, calcination temperatures ranging between 700 and 1000 °C were employed. The morphological, structural, and luminescence properties of the TiO_2_ particles, as a function of the calcination temperature and the concentration of Er_2_O_3_, are thoroughly investigated.

## 2. Results and Discussion

### 2.1. Effect of the Calcination Temperature on the Morphological, Structural, and Luminescence Properties of the TiO_2_ Particles

**Figure 1:**
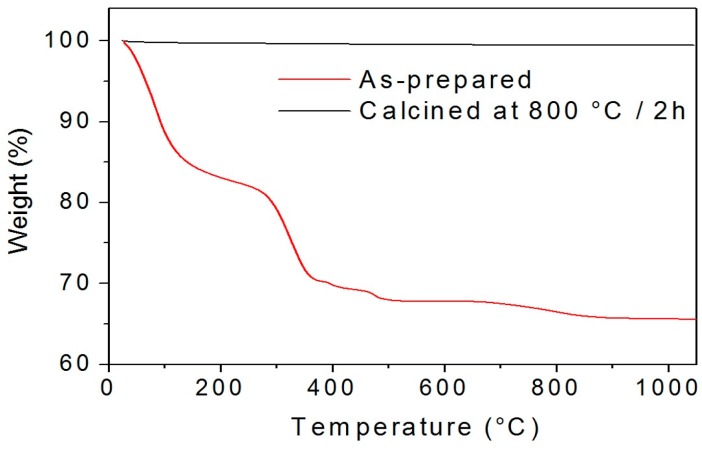
Weight loss as a function of the temperature of the 2 mol% Er_2_O_3_-doped TiO_2_ particles as-prepared and calcined at 800 °C for 2 h.

TiO_2_ powders synthesized by the sol-gel method typically result in an amorphous or poorly crystallized material. For this reason, an additional calcination step is required in order to control their crystallinity and to remove the luminescence quenching hydroxyl groups and organic residues. A thermogravimetric analysis (TGA) was performed on the synthesized particles to quantify the weight loss as a function of the temperature, and the results for the 2 mol% Er_2_O_3_-doped TiO_2_ particles as-prepared and calcined at 800 °C for 2 h are reported in Figure 1.

The curve of the as-prepared sample in Figure 1 shows several weight losses in the range between 300 and 500 °C, which could be ascribed to the removal of nitrates and acetates that are residuals from the synthesis. Additionally, the TGA curve highlights the different stages of the water removal, showing that almost no weight losses occur at temperatures higher than 850 °C. These results were confirmed by the TGA analysis performed on the same sample after calcination at 800 °C for 2 h (see Figure 1), where a weight loss of less than 0.6% was observed. Following these results, the investigation of the morphological, structural, and luminescence properties of the TiO_2_ particles doped with different Er^3+^ concentrations was carried out with the samples calcined from 700 to 1000 °C for 2 h, a range for which no presence of H_2_O is expected.

**Figure 2:**
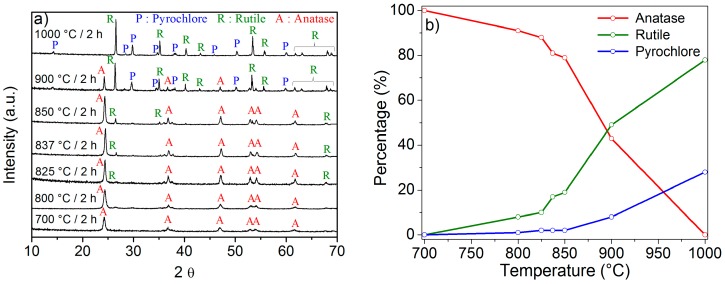
(**a**) X-Ray Diffraction (XRD) patterns of the 2 mol% Er_2_O_3_-doped TiO_2_ particles calcined at different temperatures. The diffraction peaks of anatase, rutile, and pyrochlore (Er_2_Ti_2_O_7_) are indexed in the figure as A, R, and P, respectively; (**b**) Phase composition of the TiO_2_ samples calcined at different temperatures.

The crystalline phases of the TiO_2_ particles calcined at different temperatures were identified by X-Ray Diffraction (XRD) and labelled according to the Inorganic Crystal Structure Database (ICSD) (see Figure 2a). The diffraction patterns showed the crystallographic peaks of anatase (ICSD file No. 00-021-1272) [34], rutile (ICSD file No. 00-021-1276) [34], and pyrochlore (ICSD file No. 01-073-1647).

The decrease of the Full Width at Half Maximum (FWHM) values along with an increase in the calcination temperature clearly shows that the crystallinity of the samples greatly enhanced while increasing the temperature, in agreement with [35]. The phase composition of the samples was semi-quantitatively assessed using the Reference Intensity Ratio (RIR) method. The percentages of the anatase, rutile, and pyrochlore phases present in the TiO_2_ samples are shown in Figure 2b.

The XRD pattern of the sample calcined at 700 °C shows only the anatase phase. The anatase to rutile phase transformation and the Er_2_Ti_2_O_7_ phase started to appear at 800 °C. In the case of the TiO_2_ particles calcined at 900 °C, the three phases are simultaneously present, being the rutile the major one. Lastly, at 1000 °C, the pyrochlore and rutile phases are the prominent ones. It should be pointed out that no peaks related to the Er_2_O_3_ were observed in the XRD measurements of all the calcined powders. From Figure 2a,b, it is clear that the phase composition of the particles can be tuned by varying the calcination temperature.

**Figure 3:**
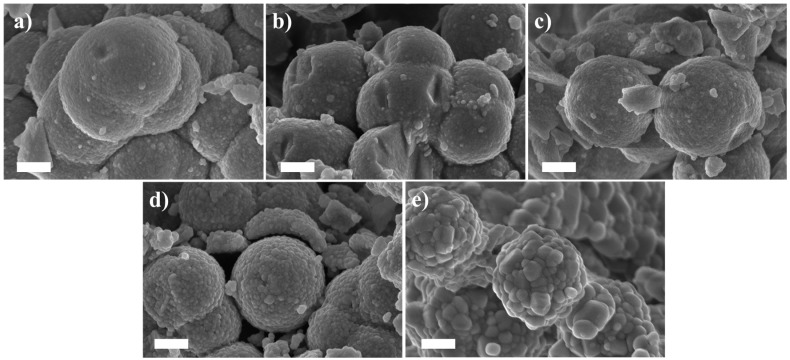
100,000× magnification Field Emission-Scanning Electron Microscope (FE-SEM) micrographs of the 2 mol% Er_2_O_3_-doped TiO_2_ particles as-prepared (**a**) and calcined at 700 (**b**); 800 (**c**); 900 (**d**); and 1000 °C (**e**) for 2 h. Scale bar equals to 500 nm.

The Field Emission-Scanning Electron Microscope (FE-SEM) pictures of the 2 mol% Er_2_O_3_-doped TiO_2_ particles prior to and after the calcination at the different temperatures are shown in Figure 3.

All the particles exhibited approximately the same spherical size, ranging from 1.3 to 1.6 μm, independently of the heat treatment, and kept their spherical shape in all the cases. Interestingly, the increase in the calcination temperature led to the growth of the crystallite size. Indeed, the texture on the particles is formed by nano-grains similar in size to those observed by Patra et al. [36]. These nano-grains possess a dimension of around 40, 50, and 60 nm for samples calcined at 700, 800, and 900 °C, respectively, while a diameter of 200 nm is reached at a calcination temperature of 1000 °C (see Figure 3).

**Figure 4:**
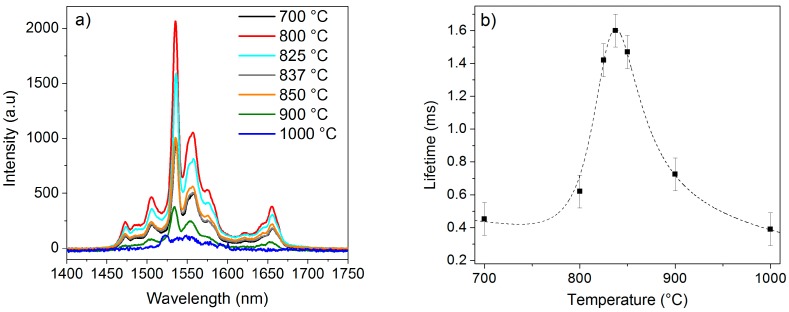
(**a**) Emission spectra of the 2 mol% Er_2_O_3_-doped TiO_2_ particles calcined at 700, 800, 825, 837, 850, 900, and 1000 °C for 2 h; (**b**) Lifetime values of the aforementioned samples. A dashed fitting line is also reported.

The photoluminescence properties of the 2 mol% Er_2_O_3_-doped TiO_2_ particles were investigated in the infrared region under an excitation wavelength of 976 nm. Figure 4a shows the fluorescence emission spectra of the aforementioned particles heat treated at different temperatures. The emission intensities were compared in this case due to the similar particles shape and the same composition of the samples.

The emission spectra corresponding to the Er^3+^:^4^I_13/2_ → ^4^I_15/2_ radiative transition show an emission band structured into different lines, with a main sharp peak centered at 1530 nm. An intense emission was observed from the sample calcined at 800 °C, whereas the samples heat treated at 900 and 1000 °C displayed a lower emission intensity.

Figure 4b shows the lifetime values of the 2 mol% Er_2_O_3_-doped TiO_2_ particles calcined at different temperatures. The lifetime values of the Er^3+^:^4^I_13/2_ level in the TiO_2_ particles calcined at 700, 800, 825, 837, 850, 900, and 1000 °C for 2 h were 0.45, 0.62, 1.42, 1.60, 1.47, 0.73, and 0.39 ms, respectively, within the accuracy of the measurement (±0.10 ms). The dependence of the lifetime on the calcination temperature could be explained by the presence of the pyrochlore phase and the reduction of the anatase phase in the particles. Surprisingly, the samples calcined at 825, 837, and 850 °C are characterized by the highest lifetime value, even though the pyrochlore phase is present in their structure (see Figure 2a,b). Nonetheless, the emission intensities of the samples heat treated at 825, 837, and 850 °C are clearly weaker than the one exhibited by the sample calcined at 800 °C (see Figure 4a). Unlike the anatase phase, the rutile phase is known to reduce the luminescence of Er^3+^ ions [37,38]. The simultaneous presence of the rutile and pyrochlore phases in the samples calcined at 825, 837, and 850 °C might thus cause a decrease in the emission intensity. In light of all these considerations, it may be concluded that the co-presence of anatase and rutile phases in the crystalline structure is essential for an optimal emission in the infrared region.

**Figure 5:**
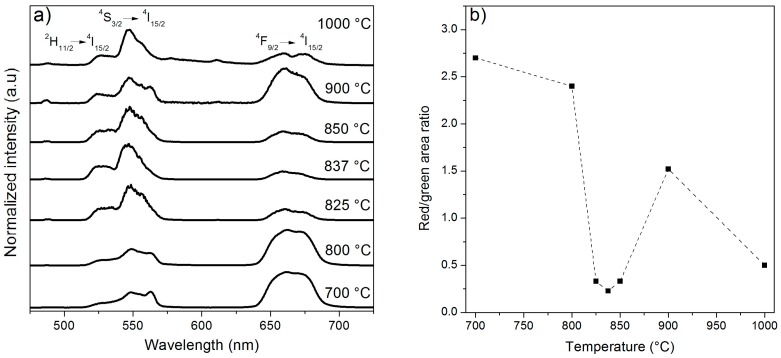
(**a**) Normalized up-conversion emission spectra of the 2 mol% Er_2_O_3_-doped TiO_2_ particles calcined at 700, 800, 825, 837, 850, 900, and 1000 °C for 2 h. All the spectra were normalized to 1 at 550 nm; (**b**) Integral area ratio of the red/green emissions of the 2 mol% Er_2_O_3_-doped TiO_2_ particles calcined at 700, 800, 825, 837, 850, 900, and 1000 °C for 2 h. A dashed line is shown as a guide to the eye.

The normalized up-conversion fluorescence spectra of the Er^3+^-doped TiO_2_ particles calcined at different temperatures are shown in Figure 5a.

The excitation wavelength of 976 nm induced a transition from the ground state, ^4^I_15/2_, to the excited level ^4^I_11/2_. Afterwards, another transition from ^4^I_11/2_ to ^4^F_7/2_ occurred. The ^4^F_7/2_ state decayed non-radiatively to the ^2^H_11/2_, ^4^S_3/2_, and ^4^F_9/2_ levels. The green emission was observed in the wavelength ranges 520–535 and 535–575 nm due to the radiative transitions from the ^2^H_11/2_ and ^4^S_3/2_ levels to the ground state, respectively. In addition, the transition from the ^4^F_9/2_ state produced a red emission in the range 640–700 nm. As shown in Figure 5a, the up-conversion emission spectra of the TiO_2_ particles calcined at 700 and 800 °C show a similar shape, and both exhibit a high intensity ratio of the red/green emissions (Figure 5b). This ratio decreased at higher calcination temperatures, being the samples calcined at 825, 837, and 850 °C the ones with the lowest red emission. Interestingly, Patra et al. [36] obtained the maximum up-conversion emission intensity with Er^3+^-doped TiO_2_ particles calcined at 800 °C, when both the anatase and the rutile phases were present. In our case, the samples calcined at 700 and 800 °C possessed the highest intensity ratio of the red/green emissions, whereas at higher temperatures the ratio considerably diminished, probably due to the presence of the pyrochlore phase.

Therefore, since the sample calcined at 800 °C showed a relatively high emission in the infrared, as well as no weight loss after the calcination, the effect of the Er^3+^ ions concentration on the properties of the synthesized TiO_2_ particles was studied only for the samples calcined at that temperature.

### 2.2. Effect of the Concentration of Er_2_O_3_ on the Morphological, Structural, and Luminescence Properties of the TiO_2_ Particles

**Figure 6:**
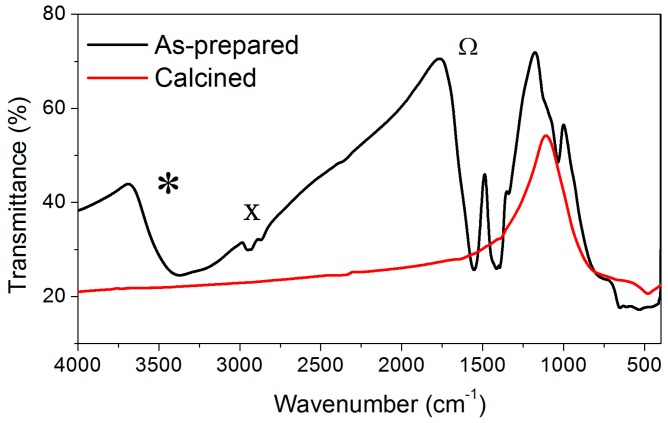
Fourier Transform-Infrared Spectroscopy (FT-IR) spectra of the 5 mol% Er_2_O_3_-doped TiO_2_ particles as-prepared and calcined at 800 °C for 2 h. *: hydroxyl groups; X: adsorbed water; Ω: O–H bending vibrations.

In order to check the possible presence of water in the calcined samples, the Fourier Transform-Infrared Spectroscopy (FT-IR) spectra of the 5 mol% Er_2_O_3_-doped TiO_2_ particles as-prepared and calcined at 800 °C for 2 h were compared in Figure 6. As can be clearly observed from the image, the typical absorption bands located at 1630, 2840, and 3430 cm^−1^ corresponding to the O–H bending vibrations, surface adsorbed water and hydroxyl groups [39], respectively, were only present in the as-prepared sample, while no presence of water was observed for the calcined sample. Similar results were obtained for the rest of the samples doped with different concentrations of Er_2_O_3_ (data not shown). Therefore, in agreement with the TGA analysis reported in Figure 1, the presence of water in the samples calcined at temperatures higher than 800 °C can be considered negligible.

**Figure 7:**
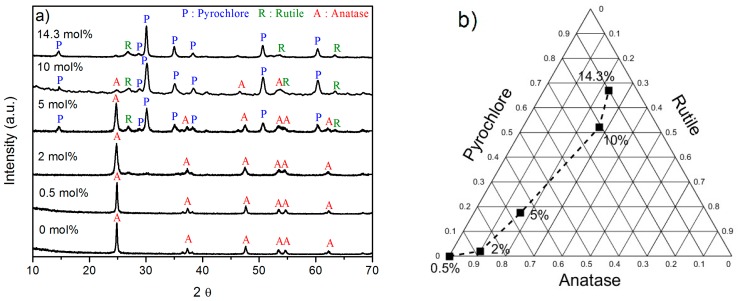
(**a**) XRD patterns of the undoped and 0.5, 2, 5, 10, and 14.3 mol% Er_2_O_3_-doped TiO_2_ particles calcined at 800 °C for 2 h. The diffraction peaks of the anatase, rutile, and pyrochlore phases are indexed in the figure; (**b**) Ternary diagram showing the proportion of the crystalline phases present in the 0.5, 2, 5, 10, and 14.3 mol% Er_2_O_3_-doped TiO_2_ particles calcined at 800 °C for 2 h.

Figure 7a shows the XRD patterns of the TiO_2_ particles calcined at 800 °C for 2 h both undoped and doped with different content of Er_2_O_3_. The phase composition of the samples was semi-quantitatively calculated using the RIR method. The ternary diagram showing the proportion of the crystalline phases present in the TiO_2_ samples doped with different concentrations of Er_2_O_3_ is shown in Figure 7b.

The undoped and the 0.5 mol% Er_2_O_3_-doped samples show the typical XRD pattern of the anatase TiO_2_. At higher concentrations of Er_2_O_3_, anatase, rutile, and pyrochlore phases are simultaneously present: at a concentration of 2 mol% the anatase phase is predominant, whereas at 5 mol% the anatase and pyrochlore phases are the prominent ones. At 10 and 14.3 mol%, the major phase seems to be the pyrochlore. Therefore, the addition of Er^3+^ ions into the TiO_2_ matrix seems to retard the anatase to rutile phase transformation. Besides, as reported in [40], the limited solubility of the Er^3+^ ions into the TiO_2_ matrix led to the formation of the pyrochlore phase, which tends to co-exist with the rutile phase. As evidenced in previous studies [33], the Er_2_O_3_ phase was not detected in the XRD measurements even with high concentrations of Er^3+^ in TiO_2_ sol-gel particles.

**Figure 8:**
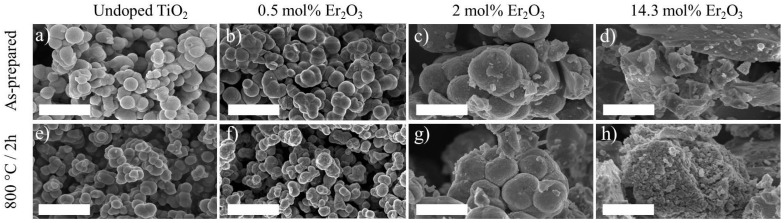
50,000× magnification FE-SEM micrographs of the undoped and 0.5, 2, and 14.3 mol% Er_2_O_3_-doped TiO_2_ particles as-prepared (**a**–**d**) and calcined at 800 °C for 2 h (**e**–**h**), respectively. Scale bar equals to 2 μm.

Figure 8 depicts the FE-SEM micrographs of the undoped and 0.5, 2, and 14.3 mol% Er_2_O_3_-doped TiO_2_ particles both as-prepared and calcined at 800 °C for 2 h.

The morphology of the particles changed when increasing the concentration of Er_2_O_3_. The undoped and 0.5 and 2 mol% Er_2_O_3_-doped particles resulted to be spherical, whereas for the higher Er_2_O_3_ concentration of 14.3 mol% the particles formed irregular-shaped aggregates. At the same time, the diameter of the spherical particles increased from 500 nm to 1.4 µm as the Er_2_O_3_ concentration raised from 0 to 2 mol% Er_2_O_3_. This might be caused by the difference in the ionic radii of Er^3+^ and Ti^4+^ ions. Indeed, the ionic radii of the Er^3+^ ions for coordination numbers equal to 6 and 8 are 0.89 and 1 Å, respectively, whereas the one of Ti^4+^ is 0.61 Å [41]. Consistently, the Er^3+^ doping affects the crystalline lattice of the anatase phase modifying the particles morphology [42]. Moreover, in agreement with the results of the XRD analysis previously reported, an increase in the Er^3+^ ions content inhibits the growth of the TiO_2_ anatase phase while increasing the rutile phase and eventually forming the pyrochlore compound. The reduction of the growth of the anatase phase and the change in the morphology of the particles with the addition of Er^3+^ is consistent with other studies [26,43].

**Figure 9:**
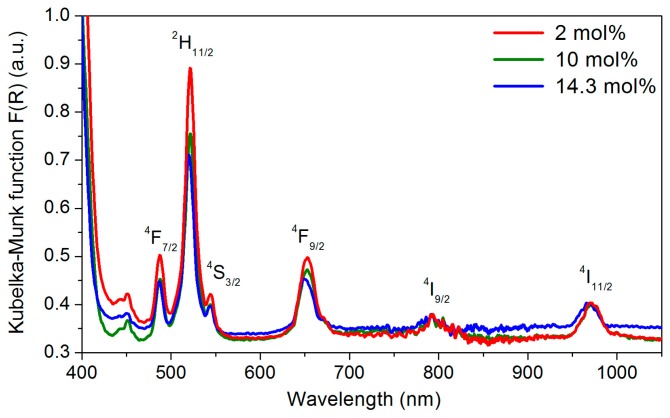
Kubelka–Munk function of the 2, 10, and 14.3 mol% Er_2_O_3_-doped TiO_2_ particles calcined at 800 °C for 2 h.

Figure 9 shows the Kubelka–Munk function of the TiO_2_ particles doped with three different concentrations of Er^3+^ and subsequently calcined at 800 °C for 2 h. The spectra exhibit several absorption bands characteristic of the Er^3+^ ion 4f-4f transitions from the ground state to various excited levels [10,44]. A broad absorption band peaked at 972 nm, corresponding to the ^4^I_15/2_ → ^4^I_11/2_ transition, can be observed. In addition, as reported in [45], the onset of the absorption spectra appears at around 400 nm, which corresponds to the anatase and rutile band gaps of 3.2 and 3.0 eV, respectively [46].

**Figure 10:**
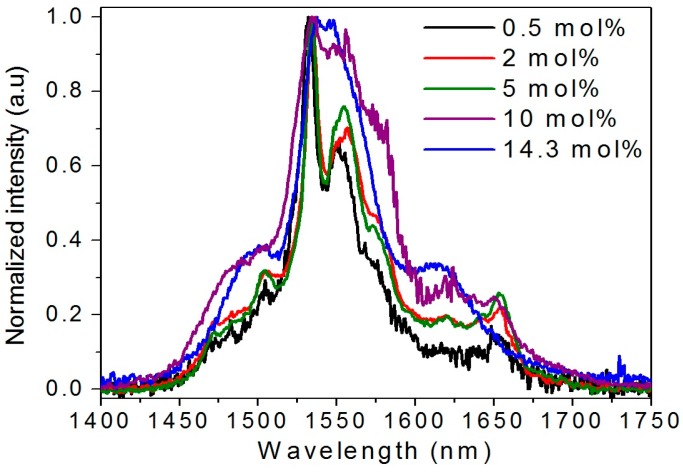
Normalized emission spectra of the 0.5, 2, 5, 10, and 14.3 mol% Er_2_O_3_-doped TiO_2_ particles calcined at 800 °C for 2 h.

Figure 10 shows the normalized emission spectra centered at around 1550 nm of the TiO_2_ particles doped with five different concentrations of Er^3+^ and subsequently calcined at 800 °C for 2 h. It should be pointed out that the intensity of the emission spectra cannot be compared due to the different morphology of the samples, although their shape gives valuable information on the particles properties.

The shape of the emission spectrum is preserved for all the samples except for the ones doped with 10 and 14.3 mol% of Er_2_O_3_, where the ^4^I_13/2_ to ^4^I_15/2_ transition peak becomes broader most probably due to the presence of the pyrochlore phase. These results are in agreement with those reported in previous studies [8], where a broadening of the peak at 1530 nm was observed for Er^3+^ doping concentrations as high as 10 and 15 mol%. Interestingly, the 10 mol% Er_2_O_3_-doped TiO_2_ particles showed the broadest spectrum, possibly due to the co-existence of the anatase and pyrochlore phases.

**Figure 11:**
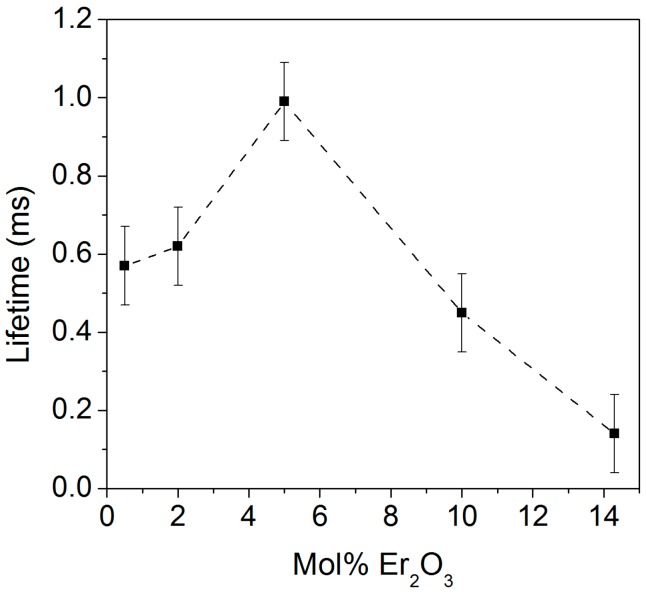
Lifetime values of the TiO_2_ particles doped with different concentrations of Er_2_O_3_ and calcined at 800 °C for 2 h. A dashed line is shown as a guide to the eye.

The fluorescence lifetime values corresponding to the intra-4f transition from ^4^I_13/2_ to ^4^I_15/2_ are shown in Figure 11. The lifetimes of the TiO_2_ particles doped with 0.5, 2, 5, 10, and 14.3 mol% of Er_2_O_3_ and calcined at 800 °C for 2 h were 0.57, 0.62, 0.99, 0.45, and 0.14 ms (±0.10 ms), respectively.

It is well-known from the literature that at high concentrations of Er_2_O_3_ the distance between the Er^3+^ ions lessens, thus leading to the formation of Er^3+^ clusters and so to shorter lifetime values [47]. Surprisingly, the 5 mol% Er_2_O_3_-doped TiO_2_ particles exhibited the highest lifetime. For this concentration, both the rutile and pyrochlore phases are present, together with the anatase phase. The presence of rutile and pyrochlore phases is thought to decrease the amount of Er^3+^ ions in the anatase phase, thus causing an increase in the radiative emission from the anatase phase as a consequence of the reduction of the quenching inside it.

**Figure 12:**
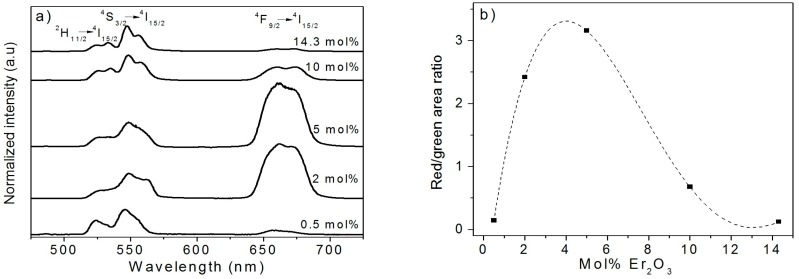
(**a**) Normalized up-conversion emission spectra of the TiO_2_ particles doped with different concentrations of Er_2_O_3_ and calcined at 800 °C for 2 h. All the spectra were normalized to 1 at 550 nm; (**b**) Integral area ratio of the red/green emissions of the TiO_2_ particles doped with different concentrations of Er_2_O_3_ and calcined at 800 °C for 2 h. A dashed fitting line is also shown.

Figure 12a illustrates the normalized visible up-conversion emission spectra of the Er_2_O_3_-doped TiO_2_ particles excited at 976 nm. The spectra were normalized to 1 at 550 nm (^4^S_3/2_ to ^4^I_15/2_ transition).

As previously explained, the intensity ratio of the red/green emissions is strictly related to the local environment of the Er^3+^ ions. Figure 12b shows an increase of the red/green emissions ratio while increasing the concentration of Er_2_O_3_ up to 5 mol%. However, for a higher Er_2_O_3_ content, the green emission is favored again. At a very low dopant concentration (0.5 mol%), the green emission is stronger than the red one because the ^4^S_3/2_ level decays radiatively to ^4^I_15/2_. Instead, at 2 and 5 mol% of Er_2_O_3_, a strong red emission resulting from the ^4^F_9/2_ to the ^4^I_15/2_ transition is observed. Patra et al. [36] have reported the increase of the ratio of the red/green emission intensities with the increasing of the Er^3+^ concentration in TiO_2_ particles doped with a low content of Er_2_O_3_. Besides, the lifetime values of the ^4^S_3/2_ level of TiO_2_ particles can diminish for higher concentrations of Er_2_O_3_ as a result of the cross-relaxation processes [48]. However, most of the studies were performed at low concentrations of Er^3+^, where no presence of the pyrocholore phase was evidenced. Surprisingly, at 10 and 14.3 mol% of Er_2_O_3_, the green emission (550 nm) arising from the ^4^S_3/2_ to the ^4^I_15/2_ transition revealed to be predominant. This strong green emission at high Er^3+^ levels is thought to be associated with the huge amount of the pyrochlore phase, which enhances the up-conversion in the green.

## 3. Materials and Methods

The following chemical precursors were used without further purification: tetra (*n*-butyl) titanate (Alfa Aesar, Haverhill, MA, USA, >99%), erbium acetate (Sigma-Aldrich, St. Louis, MO, USA, >99.9%), and ethanol (Sigma-Aldrich, St. Louis, MO, USA, >99.8%). For the synthesis of 5 g of Er^3+^-doped TiO_2_ particles containing 14.3 mol% of Er_2_O_3_ and 85.7 mol% of TiO_2_, 21.3 g of tetra (*n*-butyl) titanate were dissolved in ethanol (100 mL) and then added dropwise into a mixture of distilled water (2 mL), ethanol (100 mL), and erbium acetate (8.7 g). The process was carried out in a four-neck round-bottom flask equipped with a thermometer, a reflux refrigerator, and a magnetic stirrer. Once the addition had been completed, the precursor solution was heated at a reflux temperature of 90 °C and left under reflux for 1 day. The obtained precipitates were collected by centrifugation, washed with ethanol several times, and dried at 100 °C for 1 day. The as-prepared sample was further annealed in air at 800 °C for 2 h. The same fabrication process was followed for the 0.5, 2, 5, and 10 mol% Er_2_O_3_-doped TiO_2_ particles. The particles doped with 2 mol% of Er_2_O_3_ were heat treated in air for 2 h not only at 800 °C, but also at 700, 825, 837, 850, 900, and 1000 °C.

The thermogravimetric analysis (TGA) was performed using a Perkin-Elmer TGS-2 (PerkinElmer Inc., Waltham, MA, USA). The measurement was carried out in a Pt crucible at a heating rate of 10 °C/min, featuring an error of ±3 °C.

The Fourier Transform-Infrared Spectroscopy (FT-IR) spectra of the samples as-prepared and calcined at 800 °C for 2 h were acquired in transmission mode in the wavelength range between 400 and 4000 cm^−1^ using a Nicolet Is50 FT-IR (Thermo Fisher Scientific Inc., Waltham, MA, USA). The samples were prepared by mixing and pressing the Er-doped titania particles with potassium bromide (KBr) (weight ratio of 1:100) into disks with a thickness of 0.1 mm and a diameter of 1 cm.

The XRD analysis was performed with a PANalytical X’Pert ProMRD diffractometer (PANalytical B.V., Almelo, The Netherlands) with CuKα radiation (λ = 0.15418 nm). Data were collected with a step size of 0.02°. All XRD patterns were analyzed using X’pert HighScore Plus software (PANalytical B.V., Almelo, The Netherlands). The semi-quantitative analysis of the crystalline phases of the samples was performed using the Reference Intensity Ratio (RIR) method [49].

The morphological analysis of the samples was performed by using a Field Emission-Scanning Electron Microscope (FE-SEM, Zeiss Merlin 4248, Oberkochen, Germany) operating at 5 keV.

The optical absorption spectra were measured on the same specimens employed for the FT-IR analysis by Diffuse Reflectance Spectroscopy (DRS) using a Shimadzu UV-2600 UV-Visible (UV-Vis) spectrophotometer (Shimadzu, Kyoto, Japan). The spectra were acquired in the range between 400 and 1050 nm with a step size of 1 nm and by using barium sulfate (BaSO_4_) as a reference. The Kubelka–Munk function F(R) [50] was used to calculate the absorbance of the samples.

The emission spectra were acquired at room temperature using an excitation monochromatic light at 976 nm, emitted by a single-mode fiber pigtailed laser diode (CM962UF76P-10R, Oclaro Inc., San Jose, CA, USA).

The fluorescence lifetime values of the Er^3+^:^4^I_13/2_ energy level were obtained with laser pulses of the 976 nm laser diode, recording the signal using a digital oscilloscope (Tektronix TDS350, Tektronic Inc., Beaverton, OR, USA) and fitting the decay traces by a single exponential. The estimated error of the measurement was ±0.10 ms. The detector used for this measurement was a Thorlabs PDA10CS-EC (Thorlabs Inc., Newton, NJ, USA). The samples used for both the emission and lifetime measurements were pressed to form flat disks and placed between two transparent pure silica glasses.

## 4. Conclusions

In conclusion, both undoped and Er_2_O_3_-doped TiO_2_ particles were successfully synthesized by the sol-gel technique and their morphological, structural, and luminescence properties were thoroughly investigated as a function of the calcination temperature and the Er_2_O_3_ concentration. The surface roughness and the grain size of the particles were found to increase with the rise in the calcination temperature, while a change in the particles shape from spherical to irregular was observed upon increasing the Er_2_O_3_ doping content. Strong fluorescence in the infrared region was exhibited by the particles calcined at temperatures ranging between 800 and 850 °C, while the ones heat treated at 1000 °C showed poor fluorescence properties, thus indicating the presence of luminescence quenching induced by the formation of the pyrochlore (Er_2_Ti_2_O_7_) phase. This crystalline phase plays also an important role in the luminescence of the samples containing different amounts of Er^3+^ ions. Specifically, the particles doped with 10 and 14.3 mol% of Er_2_O_3_ showed a broadening of the emission peak associated to the ^4^I_13/2_ to ^4^I_15/2_ transition and a strong decrease in the lifetime value corresponding to the same transition. Strong up-conversion luminescence was observed in all the synthesized samples. The intensity ratio of the red/green emissions was found to change as a function of the Er^3+^ ions concentration and the calcination temperature. The highest values of this ratio were achieved for the particles doped with 2 and 5 mol% of Er_2_O_3_ and calcined at 700 and 800 °C for 2 h, respectively. The 5 mol% Er_2_O_3_-doped TiO_2_ particles calcined at 800 °C for 2 h exhibited also quite a high lifetime value corresponding to the intra-4f transition from ^4^I_13/2_ to ^4^I_15/2_, thus making them promising materials for the fabrication of infrared and up-conversion optical amplifiers and lasers.
